# Supplementation with commercially available probiotic bacterial strains reduces fat accumulation in *Caenorhabditis elegans* by regulating the lipid metabolism pathway

**DOI:** 10.17912/micropub.biology.001959

**Published:** 2026-01-05

**Authors:** Ryuichi Saito, Rika Inomata, Dian-Sheng Wang, Satoshi Shimazaki

**Affiliations:** 1 Research Division, TOA Biopharma Co., Ltd., Tatebayashi, Gunma, Japan

## Abstract

*Enterococcus faecium* T-110 (EFT110), *Bacillus subtilis* TO-A (BSTOA), and *Clostridium butyricum* TO-A (CBTOA) are probiotic bacteria used as supplements to improve intestinal symptoms in humans, chickens, pigs, cattle, and fish. However, their effects on hosts remain unclear. Our previous study showed that EFT110, BSTOA, and CBTOA extend the lifespan of *Caenorhabditis elegans* . In this study, we investigated the effects of these three strains on fat accumulation in nematodes; and observed that each bacterium significantly reduced fat accumulation by regulating gene expression in lipid metabolic pathways.

**Figure 1. Supplementation with three probiotic bacteria reduces fat accumulation in Caenorhabditis elegans:**
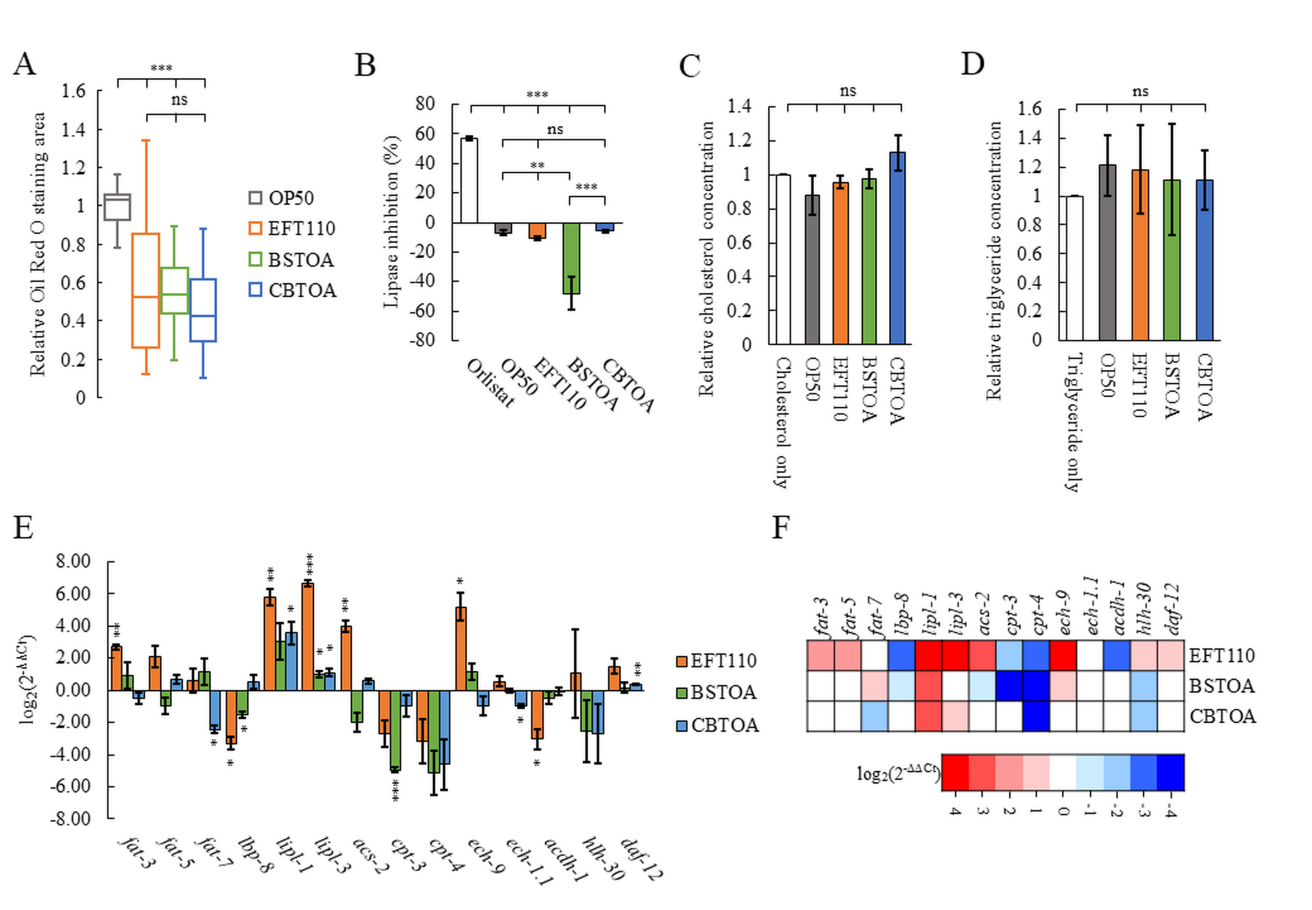
(A) Relative quantification of Oil Red O staining area in *C. elegans* fed each bacterium, normalized to nematodes fed *Escherichia coli* OP50 ( OP50 ) set as 1.0. *n* = 20. (B) Substrate was added to a mixture of porcine lipase and test samples (Orlistat, OP50 , EFT110, BSTOA, or CBTOA). After 15 min of incubation, the lipase inhibiting rate was measured. *n* = 3. (C) Cholesterol and (D) triglyceride degradation rates of each bacterium. *n* = 3. Relative concentrations of (C) cholesterol and (D) triglyceride were calculated considering the relative concentration in OP50 as 1.0. (A–D) Error bars indicate standard error (SE). Significant differences were determined using the one way ANOVA of variance with Tukey–Kramer test. (E) Expression levels of lipid-related genes in nematodes fed each bacterium, normalized to nematodes fed OP50 (set as 1.0). *n* = 3. Error bars indicate SE. Significant differences were determined using Welch's t-test. (F) Heatmap indicating expression levels. Blue and red indicate downregulation and upregulation, respectively.

## Description

EFT110, BSTOA, and CBTOA are commercially available as combined-probiotic preparation (BIO-THREE®) used since 1963 to improve intestinal symptoms in humans. These strains are used as dietary supplements and as probiotic additives in livestock and aquaculture. These strains effectively alleviate constipation, diarrhea, irritable bowel syndrome, inflammatory diseases, and infectious diseases (Chen et al., 2010, Huang et al *.* , 2014; Inatomi et al *.* , 2017; Nagamine, 2021; Shiozaki et al., 2014, Tsuda et al *.* , 2007) owing to reduced dysbiosis (Inatomi and Honma 2021; Shiozaki et al., 2014), enhanced short-chain fatty acid production (Honda et al., 2025; Inatomi and Honma 2023; Xu et al., 2021), and inhibition of inflammation-related gene expression (Takaoka et al., 2024). We previously reported that these strains extend the lifespan of *Caenorhabditis elegans* (Saito et al., 2023), but their broader health effects remain unclear. Obesity is a major risk factor for diabetes, hypertension, coronary artery disease, stroke, and cancer (Apovian, 2016), with incidence rising rapidly (Phelps et al., 2024). Interest in probiotic strategies has grown, as many studies report reduced fat accumulation by probiotics. Therefore, we hypothesized that EFT110, BSTOA, and CBTOA could reduce fat accumulation.

Under laboratory conditions, nematodes are typically fed *Escherichia coli* OP50 ( OP50 ), producing a microbiota composed only of OP50 . Nematodes have a short lifespan (approximately 15 days), and their transparent bodies allow real-time tissue imaging. Notably, over 70% of lipid metabolism genes have human orthologs (Zhang et al., 2013), and their fat droplets contain triglycerides, phospholipids, sphingolipids, and sterols, similar to humans. Thus, nematodes are widely used as well-established models in obesity research (Ashrafi, 2007). In this study, we assessed fat accumulation in nematodes ingesting EFT110, BATOA, or CBTOA; and evaluated the ability of these probiotic strains to reduce host fat storage.

After 24 h of feeding, fat accumulation was significantly reduced compared to nematodes fed OP50 (set at 1.00 ± 0.024), with relative ratios of 0.563 ± 0.077, 0.541 ± 0.039, and 0.459 ± 0.053, respectively ( Figure 1A ). Dietary fat is typically hydrolyzed by lipase into free fatty acids and monoglycerides for absorption. Although the mechanism in nematodes remain unclear, the observation that orlistat reduces fat accumulation, as in humans, suggests a similar intestinal lumen-based hydrolysis and uptake (Savova et al., 2022). Orlistat, an anti-obesity drug, inhibited porcine pancreatic lipase-catalyzed hydrolysis of p-nitrophenyl butyrate by 56.9 ± 1.31% ( Figure 1B ). In contrast, EFT110 and CBTOA showed no inhibition, while BSTOA significantly enhanced lipase activity by 47.9 ± 10.8% ( Figure 1B ).

To evaluate the fat-degrading activity of the probiotic strains, each strain was incubated for 24 h in M9 buffer supplemented with cholesterol or triglycerides. None degraded cholesterol or triglycerides under our conditions ( Figure 1C and D). These results suggest that these bacteria alone cannot degrade fats in the intestinal lumen and the host lipase is required for the fat degradation. Therefore, we hypothesized that these strains reduce fat by modulating fat synthesis, storage, or degradation pathways in nematodes. To test this, we performed RT-qPCR on 31 lipid metabolism genes in nematodes fed each strain (DuMez-Kornegay et al *.* , 2024;Karyn et al *.* , 2008; Lu et al *.* , 2022;Noble et al *.* , 2013;Nomura et al *.* , 2010; Savova et al *.* , 2022; Yue et al *.* , 2021; Yu et al *.* , 2021; Sternberg et al., 2024). Figures 1E and 1F show genes whose expression levels changed by more than two-fold (log₂ 2 ^-ΔΔCt^ >1 or <-1) or showed significant differences compared to the nematodes fed OP50 . In the EFT110-fed group, expression of *fat-3* (fatty acid desaturase, involved in fatty acid desaturation), *lipl-1* and *lipl-3* (lipase, associated with fat catabolism), *acs-2* (acyl-CoA synthetase, involved in fatty acid b-oxidation and synthesis), and *ech-9* (enoyl-CoA hydratase, involved in fatty acid b-oxidation) were significantly increased, whereas *lbp-8* (fat-binding protein, implicated in fatty acid transport) and *acdh-1* (acyl-CoA dehydrogenase, involved in fatty acid b-oxidation) were decreased. In the BSTOA-fed group, *lipl-3* increased, whereas *lbp-8* and *cpt-3* (carnitine palmitoyl transferase, fatty acid β-oxidation) decreased. Although *lipl-3* expression was higher in BSTOA fed nematodes, the fold change was <2 (log₂ 2 ^-ΔΔCt^ =0.980 ± 0.184), suggesting a limited role in BSTOA-induced fat reduction. In the CBTOA-fed group, *lipl-1* , *lipl-3* , and *daf-12* (nuclear receptor, fat metabolism) increased, whereas *fat-7* (fatty acid desaturase, fatty acid desaturation) and *ech-1.1* (enoyl-CoA hydratase, fatty acid β-oxidation) decreased. Although *ech-1.1* and *daf-12* expression was significantly changed in CBTOA fed nematodes, the fold changes were <2 (log₂ 2 ^-ΔΔCt^ =-0.955 ± 0.127 and 0.374 ± 0.026, respectively), suggesting a limited role in CBTOA-induced fat reduction. Most altered genes were linked to fat degradation, indicating these strains may promote fat reduction via lipid catabolism though the gene profiles suggest distinct mechanisms. Identifying active factors and expanding gene expression analyses may clarify the mechanisms underlying fat reduction. Moreover, as nematodes lack organs such as adipocytes, liver, and pancreas, it remains necessary to determine whether similar effects occur in other host organisms. Such validation could broaden the potential applications of these probiotics.

## Methods

***C. elegans maintenance and bacterial culture conditions***

*C. elegans* N2 Bristol and OP50 were purchased from the Caenorhabditis Genetics Center (CGC). Worms were maintained at 20°C on 60 mm nematode growth medium (NGM) agar plates (3 g NaCl, 17 g agar, 2.5 g peptone, 25 mM KPO _4_ buffer, 1 mM MgSO _4_ , 1 mM CaCl _2_ , 5 µg·mL ^−1^ cholesterol in ethanol, and 975 mL H _2_ O) seeded with OP50 . All experiments were conducted using adult nematodes 3 days post-hatching. OP50 or BSTOA were cultured in 5 mL of Luria-Bertani (1 g tryptone, 0.5 g yeast extract, 0.5 g NaCl, and 100 mL sterile water, pH 7.0) or Tryptic Soy Broth, respectively, under aerobic conditions at 37°C by shaking for 24 h. TOAT110 or CBTOA were cultured statically in 5 mL de Man Rogosa Sharpe (MRS) or gifu&nbsp;aerobic&nbsp;medium (GAM) broth, respectively, under anaerobic conditions for 24 h. Cells were harvested by centrifugation, washed three times with M9 buffer (3 g KH _2_ PO _4_ , 6 g Na _2_ HPO _4_ , 5 g NaCl, 0.02% gelatin, 1 mM MgSO _4_ , and 1 L H _2_ O), and collected as pellets.

***Oil Red O staining and quantitation***

Oil Red O staining was performed as described (Aarnio et al., 2011; Eyleen et al., 2009; Wang and Ching, 2021). Briefly, 2× MRWB (160 mM KCl, 40 mM NaCl, 357.5 mg EGTA, 266.3 mg HEPES, 50 mL H _2_ O, pH 7.4) was supplemented immediately with 0.2% β-mercaptoethanol, 2% paraformaldehyde, 1 mM spermidine, and 0.4 mM spermine, before use. Nematodes were cultured on 60 mm plates with modified NGM without peptone onto 200 mL of bacterial suspension was uniformly spread (10 mg mL⁻ ^1^ in M9 buffer), at 20°C for 24 h. After incubation, worms were washed with M9, resuspended in 100 mL M9 and 100 mL of 2× MRWB, and incubated on a rocking shaker at room temperature (RT) for 1 h. Next, the samples were washed with M9 and incubated in 1 mL of 60% isopropanol at RT for 15 min for dehydration. Subsequently, 1 mL of Oil Red O solution was added, and the nematodes were incubated on a rocking shaker at RT for 24 h. An Oil Red O solution was prepared by dissolving 125 mg Oil Red O in 41.75 mL of 60% isopropanol. Stained nematodes were placed on 2% agarose pad mounted on a glass slide and imaged using a stereomicroscope equipped with a camera (WRAYCAM-EL310; WRAYMER, Osaka, Japan). The stained regions were quantified using ImageJ software (version 1.54f). To enhance the contrast, they were inverted and split into RGB channels. The blue channel was used to set a consistent threshold for all areas based on the pixel count.

***Cholesterol degradation assay***

Cholesterol degradation was analyzed following previously described protocol (Tomaro-Duchesneau et al., 2014). Briefly, 10 mg bacterial cells and 100 mL cholesterol (10 mg mL⁻ ^1^ in ethanol) were added to 5 mL of M9 buffer and incubated with shaking at 37°C for 24 h. After incubation, 10 mL ethanol and 5 mL 33% KOH were added, vortexed, cooled to RT, and extracted with 30 mL hexane. The upper layer was collected, evaporated to dryness, and dissolved in 1 mL of ethanol. Cholesterol concentration was measured using LabAssay ^TM^ Cholesterol Kit (FUJIFILM Wako Pure Chemical Corporation, Osaka, Japan). A standard curve was generated with kit standards, and cholesterol levels in samples were calculated accordingly.

***Triglyceride degradation assay***

Triglyceride degradation was assessed using a modified method from Zhao et al. (2022). Briefly, 10 mg of bacterial pellets were incubated with 50 µL tributyrin (10 mg mL⁻¹) in 5 mL M9 buffer at 37°C with shaking for 24 h. Subsequently, 200 µL culture was mixed with 10 mL ethanol, vortexed 30 s, and centrifuged (4,000 × g, 5 min). One milliliter of supernatant was collected, and triglyceride concentration was determined using the LabAssay ^TM^ Triglyceride Kit (FUJIFILM Wako) with a standard curve from kit standards.

***Lipase inhibition assay***

Lipase inhibition was tested using a modified protocol from Núñez et al. (2023). Porcine pancreatic lipase (2.5 mg mL ^-1^ ) was prepared in Tris-base buffer (0.1 M Tris-HCl, pH7.0, 5 mM CaCl _2_ ). After centrifugation (2,000 *×g* , 3 min) 40 µL bacterial suspension or orlistat control (100 mg mL⁻ ^1^ ) was mixed with 40 µL enzyme solution on ice. Substrate solution (20 mL of 10 mM p-Nitrophenyl butyrate in Tris-base buffer) was added and absorbance (Abs) at 400 nm measured every 5 min at 37°C. Reactions without substrate served as blanks. Lipase inhibition was calculated:

Lipase inhibition (%) = [(Abs of lipase only － Abs of sample with added bacteria) / Abs of lipase only] ×100

***RNA purification and RT-qPCR analyses***

One hundred nematodes; fed bacteria for 1 day at 20°C were collected in lysis solution [0.5% Triton X-100, 0.5% Tween 20, 0.25 mM EDTA, 2.5 mM Tris-HCl, pH 8.0, 8% RNAsecure™ RNase Inactivation Reagent (Thermo Fisher Scientific Inc., WA, USA), and 1 mg mL ^−1^ proteinase K] and flash-frozen in liquid nitrogen (Jiang et al., 2019). Worm lysates were prepared by heating at 65°C for 15 min, followed by heating at 85°C for 1 min. To these lysates, 100% ethanol (2.5 times the volume of the sample) and 3 M sodium acetate (10% volume of the sample) were added, and stored overnight at −30°C. After centrifugation (14,000 *×g* , 20 min), RNA was purified, and cDNA synthesized using PrimeScript™ RT reagent Kit with gDNA Eraser (Takara Bio Inc., Kusatsu, Japan). RT-qPCR was performed with TB Green® Premx Ex Taq™ II (TaKaRa Bio) on the Quantstudio 1 real-time PCR System (Thermo Fisher Scientific Inc., WA, USA). Primers are listed in the Reagents section. Relative expression was calculated by the 2 ^-ΔΔCt^ method, normalized to four housekeeping genes ( *tba-1 , cdc-42 , eif-3 .C, pmp-3* ) (Taki and Zhang, 2013).

***Statistical analysis***

Statistical analyses conducted in R software (version 4.4.0). Statistical significance was set at *p* < 0.05. Values are annotated as ns (not significant), * *p* < 0.05, ** *p* < 0.01, or *** *p* < 0.001.

## Reagents

　Primers of each gene using RT-qPCR. Forward and reverse primers are denoted as For or Rev, respectively.

*tba-1*

For: CGTACACTCCACTGATCTCTG, Rev: CTTTGGAACGACATCTCCTCTG

*cdc-42*

For: CTATCGTATCCACAGACCGAC, Rev: CTGGATCATCCCTGAGATCG

*eif-3 .C*

For: TGCCACTGTTTCGCTCAAGAAG, Rev: GTGCATGATGAGACAGTCGGTG

*pmp-3*

For: CTACACTTTCACCGCCCAATGAC, Rev: TCCACATCCACTGTCTCCAGTTATC

*pod-2*

For: ATTGAGCAGTATTACGAGACGCAG, Rev: CATAAACGTGTTGAGCTGCTGTTC

*fasn-1*

For: GAACATCAATGTCTTCTCTGCCACC, Rev: TCCGTAGTAGAATGGTTGGTTGTC

*elo-2*

For: CATGGGCTCGTTGGTCTTTGG, Rev: GGGCGTGGAGTTTGGATGTTC

*fat-3*

For: CCACACGCAACATGACTCCATC, Rev: AGTAGTCATCGACGAGGTAAGGAAG

*fat-5*

For: GATGGGTATTCCTCCTGCACAC, Rev: CCATGAGAGGGTGGCTTTGTAG

*fat-7*

For: TGTTTCACACTTCACGCCACTTG, Rev: TGGGAATGTGTGGTGGAAATTGTG

*lbp-1*

For: GACAGCACCAAACACGAGATCAC, Rev: AGGTACTCGTAGGTCTCTTCCTTG

*lbp-8*

For: GGTGACACTTGGCATTTCAATC, Rev: AACCAAAGTGTTGTAGGATCGCTC

*lipl-1*

For: GTTGAAGCTGGAGTTTGTGACGATG, Rev: GAGTAGAAGTTCCTGCGGGTGTATG

*lipl-3*

For: TGGACTATGAACCTGCCCGAG, Rev: TGTGCTTCATGCTGTAAGTGTTCC

*atgl-1*

For: TGAAGAAACGTGCGAGTGCAAATG, Rev: GGAATATCACTGTCACCACACGTC

*acs-2*

For: CCAAAGAGACACAAGCACAGCAAC, Rev: GCTCTGGGATGTCATGGGATTTG

*acs-5*

For: CCGGATTTGAACTACTTTGCGAAGG, Rev: CTTCATCAAACAACTCGGCGGTTC

*acs-22*

For: GCCTGTGATGGATGTGGAAGATG, Rev: CCTTGACGACAATACCAGCCATTC

*acox-1.4*

For: TGATACGGATAAGATGGCGGCTG, Rev: GGTCCATGAACGGAAAGGGTTG

*ech-9*

For: GTGGAACTATGGGAAAGGCAATGG, Rev: TCCCGAGCATACATAACTTCCAAC

*ech-1.1*

For: TGCATCCAATACTTCTGCTCTTCC, Rev: TAGTCCTAGCTGAGCAGCAGTTG

*acdh-1*

For: GAATCCGAGCTTCATCCACTTGTC, Rev: CTGAGCCAGTCCAATCATTTGTGC

*sbp-1*

For: GATTGGATTGCTCGCTGGAAGTG, Rev: TGCTAGTTCCATCCGGGTCATC

*hlh-30*

For: GAGTCGCCACCAAATCACACG, Rev: GTTCGGCGATGTGATCTGCATG

*daf-12*

For: TGTTGGCTGTTCTCTTCTCTGTCC, Rev: CTGCTCTCCGAACAACGATTCC

*nhr-49*

For: AGATGACGCACCCACAAGATATG, Rev: AACTCGGAGAGCAGAGAATCCAC

*nhr-76*

For: TCGACGATATTCTGTTCTCTGCTGC, Rev: GTATAGTGCGGCAAGGGTTTCG

*nhr-80*

For: TGAGGAATTTGTGGCGCTGAAG, Rev: GTTGGGTGATTAGGTGACGTTTCG

*ser-6*

For: CTGTCCGTCAGCCAATCAAATACC, Rev: GAGTATGACAGCCAGAAGTAGGGAG

*mod-1*

For: GTGAAAGCAATGGATGTGTGGATG, Rev: CGCGTTTCTTACGCTGTTCTGAC

*daf-15*

For: CAGGAAGTAAACAACAGACAGGACC, Rev: TCGACTCGACCATCAGCATAAC

*let-363*

For: AGCTGTAAGAATCGCCGGAATTG, Rev: GTTTGGTTGACCTTCCAGTGCTTC
